# Rapid characterization of secreted recombinant proteins by native mass spectrometry

**DOI:** 10.1038/s42003-018-0231-3

**Published:** 2018-12-03

**Authors:** Gili Ben-Nissan, Shay Vimer, Shira Warszawski, Aliza Katz, Meital Yona, Tamar Unger, Yoav Peleg, David Morgenstern, Hadas Cohen-Dvashi, Ron Diskin, Sarel J. Fleishman, Michal Sharon

**Affiliations:** 1 Department of Biomolecular Sciences, Weizmann Institute of Science, 7610001 Rehovot, Israel; 2 Department of Structural Biology, Weizmann Institute of Science, 7610001 Rehovot, Israel; 3 Israel Structural Proteomics Center, Weizmann Institute of Science, 7610001 Rehovot, Israel; 4 The De Botton protein Profiling Institute of the Nancy and Stephen Grand Israel national Center for Personalized Medicine, Weizmann Institute of Science, 7610001 Rehovot, Israel

**Keywords:** Proteins, Mass spectrometry

## Abstract

Characterization of overexpressed proteins is essential for assessing their quality, and providing input for iterative redesign and optimization. This process is typically carried out following purification procedures that require pronounced cost of time and labor. Therefore, quality assessment of recombinant proteins with no prior purification offers a major advantage. Here, we report a native mass spectrometry method that enables characterization of overproduced proteins directly from culture media. Properties such as solubility, molecular weight, folding, assembly state, overall structure, post-translational modifications and binding to relevant biomolecules are immediately revealed. We show the applicability of the method for in-depth characterization of secreted recombinant proteins from eukaryotic systems such as yeast, insect, and human cells. This method, which can be readily extended to high-throughput analysis, considerably shortens the time gap between protein production and characterization, and is particularly suitable for characterizing engineered and mutated proteins, and optimizing yield and quality of overexpressed proteins.

## Abstract (web summary)

Ben-Nissan et al. present a rapid mass-spectrometry method for characterizing recombinant proteins directly from culture media. They test their method on secreted recombinant proteins from yeast, insect, and human cells, revealing solubility, molecular weight, structure, ligand binding and post-translational modifications.

## Introduction

The ability to produce recombinant proteins has revolutionized biochemistry, enabling new experimental, therapeutic, industrial and commercial applications. For example, to date about 90% of industrial enzymes used for paper, leather, detergents, textiles, food, biofuels and animal feed are recombinant proteins^1^. Similarly, recombinant protein therapeutics have increased dramatically, both in number and in frequency^2^. Since 2011, more than 60 recombinant protein drugs have been approved by the FDA, including therapies for various cancers, autoimmune and autoinflammatory diseases, infectious diseases and genetic disorders^3–5^. However, generating proteins for such applications is a highly complex process, due to their high molecular weight, three-dimensional structures, heterogeneity, and dependence on production in living cells^6^.

A critical step in the development of recombinant proteins is characterization, especially when codon optimization and protein-engineering approaches are employed. Characterizing the generated protein variants is essential for assessing their quality, and providing input for iterative redesign and optimization^7,8^. Such characterization is especially relevant when selecting the ideal host system for protein expression, optimizing product yield, or employing high-throughput investigations of lead candidates. A preliminary characterization step reduces the quantity and expense of resources wasted on inadequate protein products. It also provides a means to ensure batch-to-batch consistency. However, the characterization step is usually carried out with purified proteins, with significant costs of time and labor invested in product purification. Therefore, quality assessment of overproduced proteins with no prior purification offers a great advantage.

Recently, we described a direct native mass spectrometry approach for rapid characterization of overexpressed proteins directly from crude bacterial cell lysates, overcoming the need for protein purification^9^. In spite of this progress and the fact that prokaryotic systems are often the first-choice host for initial screening of recombinant protein expression, many proteins fail to express and fold properly in this platform^10^. Alternatively, eukaryotic secretion systems provide higher capacity for proper protein folding, assembly and post-translational modifications, in comparison to bacterial hosts^10,11^. Therefore, here, we developed a direct mass spectrometry (direct-MS) approach for the analysis of recombinant proteins secreted from yeast, insect and mammalian cells. In particular, we expressed the scaffoldin cellulose-specific carbohydrate-binding module (CBM3a) from *C. thermocellum*^12^, and serum albumin in the *P. pastoris* yeast expression system, the ectodomain of *N. albigula* Transferrin receptor 1 (TfR1) in Sf9 insect cells and several antibodies in human embryonic kidney (HEK293) cell cultures.

We show that a single measurement of the crude culture medium provides in-depth structural characterization of the overproduced protein. Specifically, the high resolution afforded by the intact protein mass measurement enables immediate assessment of sequence variations, assembly state, folding conditions, and association of ligands with the generated proteins. The method, which is suitable for high-throughput studies, also enables informative time course measurements in real time that can be used to optimize expression levels and quality. Moreover, the method considerably shortens the development timeline by enabling the selection and ranking of lead candidates directly from the culture media, avoiding the burdens associated with purifying inadequate variants.

## Results

### Direct mass spectrometry characterization of recombinant proteins in yeast

**Fig. 1:**
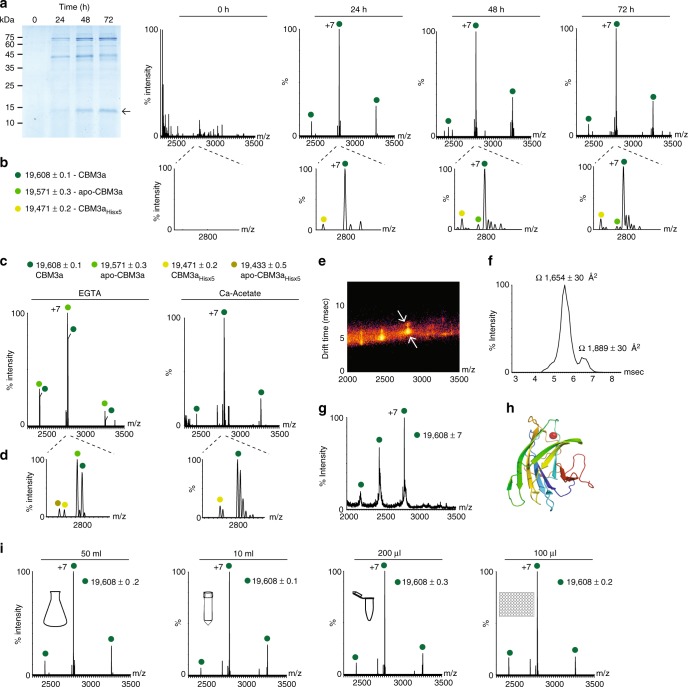
Proteins can be analyzed directly from the crude growth medium of *P. pastoris* cells. **a** SDS-PAGE and direct-MS time-course analyses of CBM3a over a period of 72 h post-induction. The band corresponding to CBM3a in the gel is denoted with an arrow. The uncropped gel image can be seen in Supplementary Fig. 11A. Mass spectra reveal that CBM3a is detected as early as 24 h after induction is initiated. **b** Enlargement of the +7 charge state at each time point (2775–2820 *m*/*z* range). The data reveal the presence of different CBM3a populations. The major population (dark green circles) corresponds to the Ca^2+^ bound form of the His×6 tagged CBM3a. Light green circles label the population of the Ca^2+^ bound form of CBM3a lacking one histidine (CBM3a_Hisx5_), and green circles label the apo-CBM3a that is not associated with the Ca^2+^ cofactor. The non-labeled minor peaks correspond to sodium and water molecule adducts. **c** Ca^2+^ binding to CBM3a was validated by incubation of the medium with the Ca^2+^ chelator EGTA (left panel). Incubation with EGTA resulted in a decrease in the intensity of the Ca^2+^-bound form of CBM3a, and a subsequent increase in the charge series of the apo-CBM3a. To recover Ca^2+^ binding to CBM3a, the medium was then incubated with calcium acetate. **d** Spectra showing the enlargement of the +7 charge states. **e** Analysis of the higher-order topology of CBM3a by native IM-MS measurements in crude medium. The IM-MS spectrum reveals the existence of two different arrival times of CBM3a, suggesting that the protein adopts two distinct conformations (marked with arrows on the mobilogram). **f** Arrival time distributions for the +7 charge states of the two populations are shown together with calculated CCS values. **g** A representative two-dimensional plot of *m*/*z* vs. intensity. **h** The crystal structure of CBM3a (PDB Entry 4JO5). **i** *P. pastoris* was induced with methanol for 24 h at decreasing culture volumes, ranging from 50 mL to 100 µL. Well-resolved charge state series of CBM3a were detected at all cell culture volumes, even at 100 µL

To investigate the applicability of the direct-MS mass spectrometry approach to eukaryotic expression systems, we initially turned to *P. pastoris*, which is considered a successful heterologous expression system for the production of recombinant biopharmaceuticals and industrial enzymes^13^. We examined the production of two proteins: CBM3a (19.6 kDa) from *C. thermocellum*^12^, and serum albumin (67 kDa). We began by monitoring the expression profiles of the two proteins by SDS-PAGE at different time points, following their induction. Twenty-four hours following methanol induction, expression of both CBM3a and albumin was detected (Fig. 1a and Supplementary Fig. 1).

To test whether we could obtain similar types of information by means of native mass spectrometry, collected aliquots were buffer exchanged into 1 M ammonium acetate at pH = 7.0, to ensure compatibility with mass spectrometry analysis. Spectra were then recorded on an Orbitrap platform^14^, without any concentration step. Highly resolved peaks with narrow charge state distributions were detected 24 h after induction initiation, with negligible contributions from background proteins, indicating that CBM3a and albumin are expressed in soluble and folded forms (Fig. 1a and Supplementary Fig. 1). From the mass measurements, we could confirm that the signal peptides of both proteins were properly cleaved. We could also determine that CBM3a is associated with one calcium (Ca^2+^) ion, as would be expected from a protein containing a calcium-binding domain^12^. In addition, we identified another minor charge series corresponding to a CBM3a species lacking one histidine residue (CBM3a_Hisx5_), from the C-terminal His×6 affinity tag designed for subsequent purification. Moreover, we noticed that at longer production times (48 and 72 h), a population of apo-CBM3a lacking the associated Ca^2+^ ion (apo-CBM3a) is detected, suggesting the exhaustion of calcium from the growth medium, and the need for supplementation (Fig. 1b). Minor peaks of the slightly larger populations, which correspond to sodium and water adducts, were also detected. Overall, the spectra acquired directly from the growth medium revealed the expression states of both CBM3a and albumin, providing useful information for optimization of growth conditions.

To confirm that Ca^2+^ ions are indeed associated with CBM3a, we treated the growth medium with 50 mM EGTA, a calcium chelating agent. The resulting spectrum indicated the appearance of additional charge states, corresponding in mass to the apo form of the protein (Fig. 1c, d). Such populations were detected both for the full-length protein and the His truncated variant (CBM3a_His×5_). Adding 0.5 mM calcium acetate to the treated medium shifted the protein back to the holo, Ca^2+^ bound state (Fig. 1c, d), confirming that calcium is, indeed, a CBM3a-associated cofactor. Hence, cofactor identification can be performed from crude samples, eliminating need for isolation and purification steps.

Next, we wished to examine whether direct mass spectrometry analysis of secreted proteins can also be expanded to include ion-mobility mass spectrometry (IM-MS) measurements, which provide information on the higher-order structure of the protein^15,16^. In an ion-mobility experiment, the time it takes a protein to traverse a weak electrical gradient in a gas-filled chamber is measured. Drift times are then converted to collision cross-sections (CCS), reflecting the overall shape of the protein^15,16^. The resulting data indicated that despite the presence of contaminant proteins within the culture medium, well-resolved spectra could be collected for CBM3a using the Synapt G2 instrument (Fig. 1e–g). Two distinct conformations were detected for each charge state. The predominant species gave rise to a CCS value of 1654 ± 30 Å^2^. Theoretical CCS for CBM3a, calculated by the projection approximation algorithm, based on the crystal structure (PDB Entry 4JO5) (Fig. 1h), yielded a value of 1489 Å^2^, which fits well with the measured CCS value, given that the projection approximation algorithm used to calculate this value is known to underestimate CCS values by ∼13%^17^. We noticed that in all measurements, which were performed under gentle conditions such that structural perturbations are minimized, a second, minor drift-time population was observed (Fig. 1e, f). This population (15%) displayed a CCS value of 1889 ± 30 Å^2^, possibly reflecting the existence of a more extended conformation. Direct-MS ion-mobility measurements can therefore reflect the higher-order shape of unpurified proteins.

We then aimed to determine the minimal culture volume sufficient for direct-MS analysis. In order to achieve this we induced *P. pastoris* at decreasing amounts of culture volumes, starting from a 50 mL culture in a 250 mL flask, through a 10 mL culture grown in a 50 mL tube, a 200 µL culture in a 1.5 mL tube, down to a 100 µL culture in a 96-well plate format. As can be seen in Fig. 1i, well-resolved charge series of CBM3a with high signal-to-noise ratios were obtained throughout the decrease in culture volume. Even in the 100 µL cell culture grown in a 96-well plate, charge states attributed to CBM3a could be clearly detected. Given this observation, we anticipate that the direct MS approach can be scaled up for high-throughput screening.

### Characterization of overproduced proteins in insect cells

We continued by examining whether the direct-MS strategy may be applied to protein production characterization in insect cells, a system that produces proteins that are functionally similar to the native mammalian proteins, and can be readily adapted to high-density suspension cultures for large-scale expression^18^. We inoculated Sf9 cells with baculoviruses containing an expression cassette for the production of the ectodomain of *N. albigula* TfR1, fused to the N-terminal signal peptide gp67, and to a C-terminal His×6 tag. The viruses also included an expression cassette for the green fluorescent protein (GFP), which is used as a reporter for infection efficiency.

We began by collecting the culture medium at different time points post-virus infection, in order to determine the protein expression profile. SDS-PAGE analysis indicated that 48 h after infection, TfR1 is barely detected, but it can be clearly seen after 72 h (Supplementary Fig. 2). Similarly, direct-MS analysis from crude growth media revealed the production of TfR1 72 h following virus infection, whereas GFP was already detected after 24 h. Notably, GFP accumulates intracellularly in cells, and its presence in the growth medium is attributed to ongoing cell death, which causes the cellular content to spill into the medium. As evidenced by SDS-PAGE and native mass spectrometry analyses, 96 h after inoculation, the major protein in the growth medium was GFP, though TfR1 was still clearly detected. At this time point, the growth cultures are exhausted and more pronounced cell death occurs, resulting in the accumulation of high levels of GFP in the medium. Thus, direct MS enables simple, informative time course measurements that can be amenable to optimizing production yields.

**Fig. 2:**
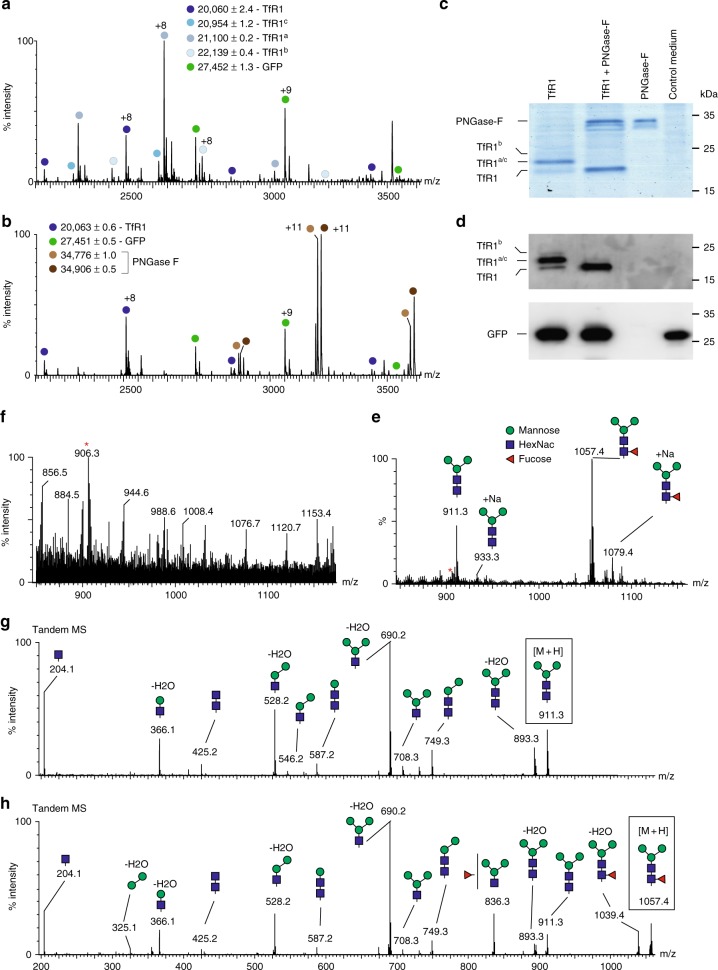
Multiple forms of TfR1 are produced in Sf9 cells. **a** Direct-MS analysis of secreted TfR1 from insect cell growth medium reveals four different populations of the protein. The major population corresponds in mass to the protein, modified with GlcNac_2_Man_3_Fuc_1_ (TfR1^a^), leading to a 1039 Da shift in mass. In addition, three other populations of TfR1 can be detected, corresponding in mass to the intact protein (TfR1), the protein labeled with two GlcNac_2_Man_3_Fuc_1_ hexasaccharides (TfR1^b^), as well as a population labeled with the pentasaccharide GlcNac_2_Man_3_ (TfR1^c^). The charge state series labeled with green circles represents GFP, which is accumulating intercellularly, but is present due to cell death, and spillage of the cellular contents into the growth medium. **b** In order to strip the glycosylations from TfR1, we treated the growth medium with PNGase F. Direct-MS analysis indicated that after 15 min of incubation, only charge state series corresponding to the unmodified form of the protein remained, while the glycosylated TfR1 populations disappeared. The series labeled with brown circles represent PNGase F. **c** SDS-PAGE and (**d**) Western blot analysis reveal the different glycoforms of TfR1 prior to and following incubation with PNGase F. The blots in **d** were reacted with a His Probe HRP-Conjugate to detect TfR1 and an anti-GFP antibody. The uncropped gel and blot images can be seen in Supplementary Fig. 11B-C. **e** Direct-MS spectra, focused on the low m/z scale, were acquired, following the addition of PNGase F to the growth medium. Two major glycans were detected, corresponding in mass to protonated GlcNac_2_Man_3_Fuc_1_ and GlcNac_2_Man_3,_ respectively, with a free reducing end. These oligosaccharides were not detected in the medium prior to PNGase F treatment (**f**). A background peptide from the growth medium is labeled with a red asterisk. **g**, **h** MS/MS analysis of the two glycans validated their assignment. Each glycan was selected within the quadrupole mass filter, and subjected to MS/MS fragmentation. The selected glycan is enclosed within a box, and the various ion fragments generated are annotated

Mass analysis of the spectra enabled the identification of multiple TfR1 forms (Fig. 2a). The most abundant population corresponds to the intact protein after cleavage of the signal peptide and modification by glycosylation. The 1039 Da shift in mass suggests that the glycoform is a hexasaccharide with a GlcNac_2_Man_3_Fuc_1_ (TfR1^a^) composition, the most common N-linked core glycan in insect cells^19^. In addition, we identified a population with two GlcNac_2_Man_3_Fuc_1_ glycosylation sites (TfR1^b^), as well as a population of TfR1 modified with a pentasaccharide (GlcNac_2_Man_3_), which is missing the fucose moiety (TfR1^c^). We also assessed the relative abundance of the differentially modified glycoforms, indicating that 65% of the protein is modified with one GlcNac_2_Man_3_Fuc_1_, while 10 and 7% are modified with two GlcNac_2_Man_3_Fuc_1_ or one GlcNac_2_Man_3_, respectively_._ Though relative quantitation for the glycoforms can be evaluated by SDS-PAGE analysis (Fig. 2c), we were not able to distinguish between the penta- and hexa-oligosaccharides.

To validate that the molecular heterogeneity is indeed caused by glycosylations, we treated the growth medium with the deglycosylating enzyme PNGase F, which removes N-linked oligosaccharides at the asparagine linkage^20^. The enzymatic removal of the glycans gave rise to a simplified spectrum of TfR1, consisting solely of the unmodified protein (Fig. 2b). The PNGase F enzyme itself, as well as GFP, were also detected in the spectrum (Fig. 2b). SDS-PAGE followed by Western blot analysis confirmed that the heterogeneity of TfR1 results from glycosylation (Fig. 2c, d).

To characterize each glycoform in detail, we treated the culture medium with PNGase F, and recorded spectra of the low *m*/*z* region (1500 > *m*/*z*). The data indicated the presence of two glycoforms, that in accordance with their measured masses of 1057 and 911 Da, are likely to be protonated GlcNac_2_Man_3_Fuc_1_ and GlcNac_2_Man_3_, respectively, containing a free reducing end (Fig. 2e). In a control measurement of the same medium prior to PNGaseF treatment, these glycans were not detected, suggesting that they indeed originate from TfR1 (Fig. 2f). We then applied the tandem mass spectrometry (MS/MS) approach, in order to further validate the composition of the two glycans. Accordingly, GlcNac_2_Man_3_Fuc_1_ and GlcNac_2_Man_3_ were isolated and collisionally activated to induce their fragmentation. Assignment of the fragments of both the glycans confirmed their structural composition (Fig. 2g, h; Supplementary Table 1). Hence, direct-MS analysis enabled glycosylation analysis directly from growth media, overcoming the need for purification and liquid chromatography/mass spectrometry measurements.

### Analysis of antibodies from crude media of human cell lines

Our next goal was to examine proteins expressed in a mammalian cell culture system. We therefore turned to HEK293 cells, which are frequently used in the manufacture of proteins for therapeutic and for industrial purposes^21^. Initially, we co-transfected adherent HEK293 cells with plasmids for the expression of light and heavy chains of the 289 antibody, a monoclonal antibody that recognizes the envelope glycoprotein 1 from the Junin mammarena virus. We then grew the cells for three days in a medium containing a reduced amount of serum (2% instead of 10%), to decrease signal masking by the bovine serum albumin. After concentrating the growth medium by a factor of 10, and exchanging the buffer into volatile ammonium acetate, a nearly baseline resolution spectrum of the secreted antibody was acquired on the Orbitrap instrument^14^ (Supplementary Fig. 3A). The measured mass and the low number of charge states indicated that a folded antibody is produced. Moreover, the 2925 Da mass deviation between the expected and measured mass is likely due to two G0F modifications, corresponding to core-fucosylated glycoforms (GlcNac_4_Man_3_Fuc_1_), one of the major modifications of recombinant antibodies^22–24^.

To overcome the need for sample concentration, and possible inflicted stress owing to the reduced serum levels, we turned to suspension-grown HEK293F cells, which are cultured in a chemically defined protein-free medium. We co-transfected cells with plasmids for the expression of light and heavy chains of the Mut23 antibody, which recognizes a sugar epitope. After 5 days of culture, we collected the medium, and acquired data directly after performing a buffer exchange step into a mass spectrometry-compatible solution. Under these growth conditions, no concentration steps were required, and a nicely resolved mass spectrum of the intact antibody could be obtained (Supplementary Fig. 3B). The data indicate that the presence of intact antibodies can be detected, as in the case of the 289 and Mut23 antibodies, with no free heavy or light chain ions present, demonstrating efficient antibody assembly.

**Fig. 3:**
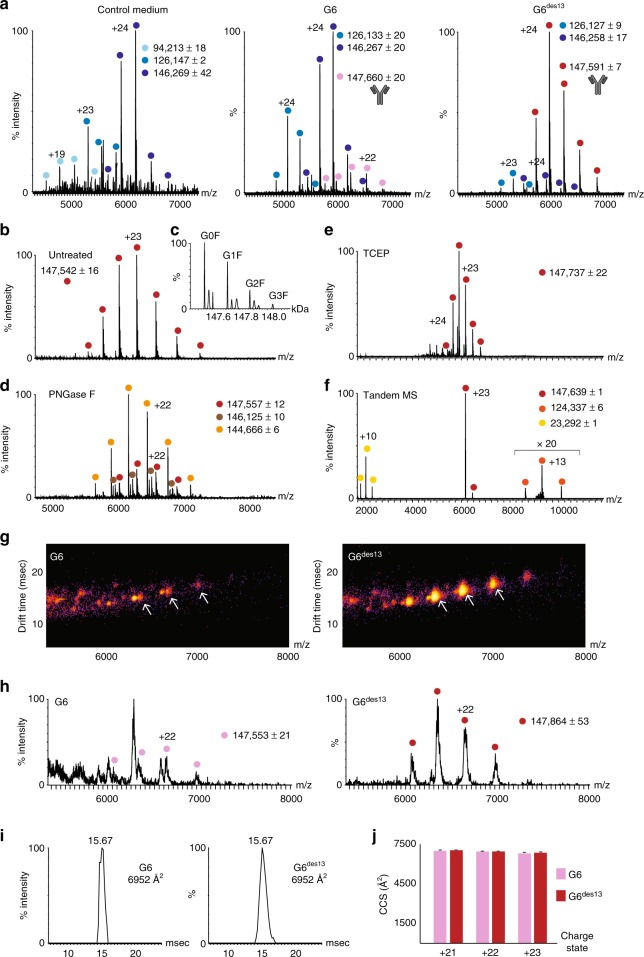
Rapid characterization of recombinant antibodies, directly from the growth medium of secreting human cells. **a** Expression levels of secreted antibodies were evaluated by direct-MS. Suspension-grown HEK293 cells were transfected with plasmids for expression of light and heavy chains of chimeric antibodies. Growth media were collected and analyzed after 5 days. In comparison, a spectrum obtained from medium of control, non-expressing cells is shown, where various background proteins were detected. The results show that the G6 antibody accumulates to low levels, below the intensity of the background proteins, whereas the designed G6^des13^ antibody accumulates to higher levels, and appears as the main charge envelope in the spectrum, due to the improved design. The antibody cartoon labels the antibodies measured mass. **b**–**d** Glycan analysis of unpurified antibodies in the growth medium. The high resolution spectrum of the G6^des13^ antibody was measured before (**b**) and after (**d**) PNGase F treatment of the growth medium. **c** Deconvolution of the spectrum of the untreated antibody revealed four major glycoforms. Although minor subpopulations of partially or fully glycosylated antibodies were detected in the treated sample, comparison of the mass of the modified antibody to that of the deglycosylated form enabled us to determine that the core glycan is G0F, and the other glycans differ by the addition of a galactose molecule (G1F, G2F, and G3F). **e**, **f** Reduction of antibodies in the growth medium enabled us to liberate their light chains. **e** The G6^des13^ antibody was reduced with TCEP, enabling the dissociation of the light chain during tandem MS analyses (**f**). **g**–**j** Analysis of the higher order topology of antibodies by whole range IM-MS measurements in crude media. Three-dimensional mobilograms (**g**) and their representative mass spectra (**h**) were measured for the G6 and G6^des13^ antibodies, directly from the crude growth media. **i** Arrival time distributions and calculated CCS values for the +22 charge states of both antibodies, measured at wave height of 18 V. **j** Results indicate that the CCS values of the two antibodies are essentially identical, and that the mutations incorporated within G6^des13^ did not modify the overall shape of the antibody. Standard deviations are shown. The original mean source data is shown in Supplementary Data 1

Next, we explored the applicability of this direct-MS approach for assessing the quality of designed versions of proteins. We examined two unrelated antibodies, targeting the human vascular-endothelial growth factor (VEGF), termed G6^25^, the anti-lysozyme antibody D44.1^26^, and their designed variants, G6^des13^ and D44.1^des^, respectively, specifically designed for increased stability, aggregation resistance, and expression yields. We began by recording spectra of the growth medium from non-expressing cells, which indicated the presence of several well-resolved charge state series between 4000 and 8000 *m*/*z*, reflecting the presence of a background signal (Fig. 3a). In attempt to identify the nature of these proteins, we performed MS/MS and pseudo-MS^3^ top-down analyses (Supplementary Fig. 4). MS/MS analysis indicated that the largest charge state series, with a mass of 146,269 Da corresponds to a tetramer, composed of monomers of 36,549 Da. MS^3^ activation and top-down analysis^14^, revealed that this protein is lactate dehydrogenase B chain, a cytosolic enzyme known to leak into the growth medium in cell cultures due to loss of cytosolic membrane integrity of damaged cells during necrosis^27,28^. Similar to the presence of cytosolic GFP in insect cell cultures (Supplementary Fig. 2), lactate dehydrogenase can serve as an indicator for the level of cell death during extensive culturing of mammalian cells.

Analysis of medium from cells secreting the G6 antibody revealed that, in addition to the background medium proteins, a relatively low-intensity charge state series, corresponding in mass to the intact, modified antibody, is detected. A different scenario was observed for the designed version of the antibody, G6^des13^. In this case, the major charge-state envelope corresponded to the designed antibody, clearly demonstrating that the modified antibody exhibits higher expressibility, compared to G6 (Fig. 3a). Similar results were obtained for D44.1 and D44.1^des^, indicating that the expressibility of the designed proteins was improved (Supplementary Fig. 5).

N-glycosylations are the most common type of post-translational modification on antibodies, and contribute to their structural characteristics by potentially modulating their function and pharmacokinetic properties^29–31^. Indeed, we noticed that all analyzed antibodies exhibit a ~3 kDa shift between their measured and calculated mass, suggesting that they are glycosylated (Fig. 3b, c, Supplementary Fig. 6). To validate this assumption, and test whether it is possible to deglycosylate antibodies directly in crude media, we treated the media with PNGase F. Molecular mass shift of spectra recorded after PNGase F treatment confirmed the release of glycans from the antibodies. (Fig. 3d; Supplementary Fig. 6). Mass measurement of the deglycosylated G6^des13^ antibody indicated that the two heavy chain C-terminal lysines are clipped (−128 × 2 Da), and that it bears 14 disulfide bridges (−28 Da). Calculating the mass difference between the deglycosylated G6^des13^ antibody and its main glycosylated species enabled us to conclude, with an accuracy of 7 ppm, that the major glycosylated form is that of a G0F (GlcNac_4_Man_3_Fuc_1_) structure on each heavy chain^22–24^ (Fig. 3c, d). In addition, three glycoforms with the characteristic 162 Da spacing, corresponding to galactose, have been defined, indicative of G1F, G2F, and G3F species, with one, two and three galactose molecules, respectively. Examination of the low m/z region of the spectrum prior to and following PNGase F treatment indicated the appearance of the two main glycans; namely, the sodiated and non-sodiated G0F (GlcNac_4_Man_3_Fuc_1_) and G1F (GalGlcNac_4_Man_3_Fuc_1_), species (Supplementary Fig. 7), which were only detected in the spectrum of the treated sample. Overall, these results suggest that isolation and purification of produced antibodies is not necessarily a prerequisite for high-resolution native mass spectrometry analysis.

Full characterization of antibodies includes the analysis of the composing heavy and light chains that are generated by reduction of the disulfide bonds^20,32^. To assess the feasibility of in-medium reduction, we added the TCEP reductant to the growth medium of the G6^des13^ antibody, prior to mass spectrometry recording (Fig. 3e). MS/MS experiments were then performed, in which a single peak corresponding to the intact antibody was isolated. The ions were then subjected to collisional activation in the higher energy collision-induced dissociation (HCD) cell, liberating the light chain from the antibody as it is no longer covalently attached to the heavy chain (Fig. 3f). The measured mass of the dissociated light chain was smaller than its theoretical mass (23,296 Da) by 4 Da, indicating that the TCEP treatment did not reduce the two intermolecular sulfur bridges within the light chains. In addition, the close agreement between the measured and calculated mass of the light chain implies that the light chains are not glycosylated, but rather the heavy chains are modified with glycans. The fact that heavy chains were not detected under any of the tested reduction experiments can be attributed to low reduction efficiency of the hinge region, possibly due to the structural properties of the antibody backbone scaffold. Similar results were obtained for D44.1 and its designed counterpart, D44.1^des^ (Supplementary Fig. 8).

The baseline-resolved, direct-MS spectra encouraged us to explore whether we could also characterize the antibodies produced directly from the crude media, by means of IM-MS. Using this approach, we first collected IM-MS data for D44.1^des^, using the Synapt G2 instrument. The results indicate that despite the impurity of the sample, well-resolved IM-MS plots could be generated (Supplementary Fig. 9). The intact mass of the antibody and its corresponding CCS value could easily be extracted, emphasizing not only the additional experimental input yielded by this method, but also the fact that the direct-MS approach can be applied on various high-mass mass spectrometry platforms.

We next explored the G6 and G6^des13^ pair of antibodies, examining whether the introduced mutations altered the overall structure of G6^des13^, in comparison to G6. IM-MS analysis demonstrated that, similar to the measurements conducted on the Orbitrap platform, the major charge series in the crude G6 sample corresponded to background medium proteins; nevertheless, the antibody signals could be clearly identified (Fig. 3g, h). In the G6^des13^ sample, the most intense charge envelope corresponded to the antibody, while the medium protein signals were very low, supporting the view that G6^des13^ exhibits a gain in expressibility. Moreover, the CCS values of G6 and G6^des13^, averaged over 3 wave heights and 3 charge states were calculated to be 6765 ± 6 Å^2^ and 6745 ± 3 Å^2^, respectively, suggesting that the two antibodies adopt similar conformations, and that the incorporated mutations did not alter the structural compactness of G6^des13^ (Fig. 3i, j). Taken together, we found that direct-IM-MS measurements can therefore reflect the higher-order shape of unpurified antibodies.

### Sensitivity evaluation of the direct-MS approach

**Fig. 4:**
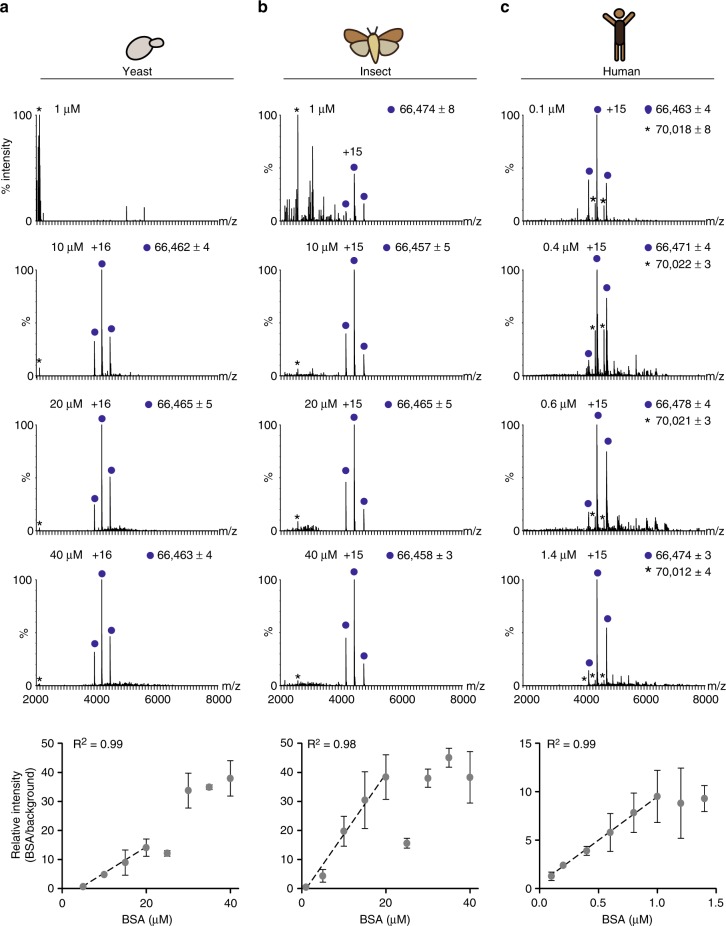
Changes in protein abundance can be monitored in crude media. A series of measurements with different concentrations of externally-added BSA, were performed in growth media from yeast (**a**), insect (**b**), and human (**c**) cell cultures. The intensity ratio between BSA (dark blue circles) and background reference peaks (labeled with asterisks) was plotted against known BSA concentrations (bottom graphs). A linear trend was found for BSA concentrations of up to 20 µM in yeast and insect cell cultures, and up to 1.4 µM in human growth media. Trend lines and R-squared values are shown. Error bars represent standard deviations of three measurements. The original mean source data is shown in Supplementary Data 2

Finally, we examined the sensitivity of the method by assessing the limit of detection of serially diluted yeast, insect and human cultures, initially supplemented with 20 µM bovine serum albumin (BSA) (Supplementary Fig. 10). The measured spectra indicated that by diluting the effective culture volume with ammonium acetate, high quality spectra of BSA can be recorded even for concentrations less than 100 nM (Supplementary Fig. 10). In addition, we examined whether it is possible to monitor changes in protein abundance within the crude media. To achieve this, a series of spectra were recorded using yeast, insect and human cultures supplemented with increasing concentrations of BSA. Figure 4 shows representative mass spectra of the three different growth media, obtained using elevated BSA concentrations. The relative abundance of BSA was then estimated in respect to selected background peaks whose intensity remained unchanged throughout the analysis. For each mass spectrum, the intensity ratio of BSA and the selected reference peaks was calculated and plotted against known BSA concentrations (Fig. 4 bottom). A linear trend line was observed for all three expression systems, however, the linearity range differed. We noticed that the range of linearity is dependent on the highest concentration level of BSA that can be quantified, which is limited by the detection of the reference peak. The low concentration of protein background in human culture (5 ng/ml), in respect to yeast and insect cells growth media (133 ng/mL and 88 ng/mL, respectively), reduces the quantification range, as seen in Fig. 4, yet, it enables the detection of low expression levels of proteins. Taken together, the data indicates that for a specified range, it is possible to measure dynamic changes in protein levels directly from culture media.

## Discussion

Here, we report a method for rapid characterization of secreted recombinant proteins, directly from crude growth media. Based on native-mass spectrometry, this method enables the assessment of multiple protein attributes in a single spectrum. We demonstrate that the expression, solubility and assembly state of overproduced proteins can be determined, as well as their associations with cofactors. The mass accuracy afforded by this strategy enables not only the identification of post-translationally modified species, but also the existence of differential modifications, and the distribution of the different forms. We also show that the overall structure of the unpurified overproduced proteins can be defined.

Here, we have focused on recombinant proteins produced in yeast, insect, and human cells; however the method can be expanded to include many other expression platforms that overproduce secreted proteins, such as Chinese hamster ovary cells (CHO)^33^ and murine myeloma cell lines (GS-NS0)^10,34^. The fundamental requirement for this approach, however, is that the overexpressed target becomes the dominant protein in the growth medium. Consequently, the in-depth structural characterization afforded by native mass spectrometry analysis can be performed. Moreover, it should be noted that the strategy is not dependent on a specific mass spectrometer platform; rather, it is a general approach that can be applied on either Orbitrap or QTOF-based platforms that provide extended mass range^35–37^.

A key to successful production of recombinant proteins for both industrial and pharmaceutical applications is to achieve maximum productivity at a reasonable cost^10,38^. Therefore, a screening program is generally performed to identify high-producing cells, optimize genetic and engineered constructs, and improve media formulation. We anticipate that the method outlined here will facilitate this labor-intensive and costly process, by overcoming the need for protein purification prior to characterization, and by providing, in a single step, in-depth quality assessment of the protein produced. In addition, the minimal amount of sample required, coupled with the simplicity and feasibility of the approach across different platforms, also make it suitable for high-throughput screening procedures that may further reduce the time gap between production and characterization.

The ability to monitor, in real time, recombinant protein production is a valuable feature of this strategy. Therefore, we expect that further extension of this method will involve its coupling to microfluidics systems that will enable continued analysis of cultures grown in bioreactors. Such analyses will make it possible to capture optimal harvesting times, preventing misfolding and proteolysis, and determine the necessity of cofactor supplements to ensure proper folding and assembly. Such knowledge will improve both yield and quality of the recombinant proteins produced.

## Methods

### Plasmids

Yeast: CBM3a, the cellulose-specific carbohydrate-binding module of the scaffoldin protein from *C. thermocellum*^12^, was cloned into the yeast expression vector pPIC9K (Thermo Fisher Scientific) under the regulation of the methanol inducible promoter of the *Alcohol Oxidase 1* gene. The gene was fused to the N-terminal α-factor secretion signal and a C-terminal His_×6_ tag. Genomic integration into *P. pastoris* was performed at the *alcohol oxidase 1* locus, according to the manufacturer’s instructions.

Insect cells: A modified monomeric ectodomain of the *N. albigula* Transferrin Receptor 1 (TfR1), was cloned into the pAcGP67-B vector, under the regulation of the polyhedrin promoter. The gene was fused to the N-terminal signal peptide gp67, and to a C-terminal His×6 tag. Sf9 cells were co-transfected with 0.4 μg of the pACgp67b vector and a 0.25 μg linearized baculovirus genome (ABvector) using EscortIV transfection reagent (Sigma). Four days later medium was collected from transfected cells (P_0_), and was used to infect fresh Sf9 cells for amplification (P_1_–P_2_).

Human cells: The variable regions of the heavy and light chains of several synthetic chimeric antibodies were cloned separately, upstream of the IgG1 human Ab scaffold, into p3BNC plasmids containing the human IgG secretion signal peptide. Antibodies used in this study were: (a) Antibodies G6 and G6^des13^, against human VEGF. The G6 antibody is based on the clinical anti-VEGF antibody avastin. The difference between G6 and its designed variant G6^des13^ are six amino acids in the interface between the light and heavy chains; (b) Antibodies D44.1 and D44.1^des^, against lysozyme. The difference between D44.1 and its designed variant D44.1^des^ are eight amino acids in the interface between the light and heavy chains; (c) Antibody 289, recognizing the envelope glycoprotein 1 from the Junin mammarena virus; (d) Antibody Mut23, recognizing a mouse sugar epitope.

### Cells and growth conditions

Yeast: CBM3a was expressed in *P. pastoris* GS115 cells. Serum albumin harboring its native secretion signal was expressed in *P. pastoris* GS115/His^+^ Mut^S^. Cells were grown in Buffered Glycerol-Complex Medium and Buffered Methanol-Complex Mediumat 30 °C, at 220 rpm. A single colony was transferred into 10 mL of Buffered Glycerol-Complex Medium, and grown until the optical density reached 4 at 600 nm (equivalent to 2 × 10^8^ cells/mL). Cultures were centrifuged, and the cell mass was collected and resuspended in 50 mL Buffered Methanol-Complex Medium. Expression was induced by the addition of 0.5% methanol every 24 h throughout the entire protein production period.

For minimal culture volume analysis, cells resuspended in Buffered Methanol-Complex Medium were divided into different culture volumes: 50 mL in a 250 mL flask, 10 mL in a 50 mL tube, 200 µL in a 1.5 mL tube, and 100 µL in a 96-well plate. Cells were grown as detailed above, except for the 96-well plate, which was covered with an AeraSeal™ breathing cover (Excel Scientific) and placed in a vibrating incubator (Heidolph Titramax 1000) at 30 °C, at 650 rpm. Growth media were analyzed 24 h post-methanol induction.

Insect cells: TfR1 was expressed in baculovirus-infected Sf9 cells. Cells were grown in ESF921 medium (Expression Systems) at 27 °C, at 140 rpm. Cultures (30 ml) containing 1.8 × 10^6^ cells/mL were infected with 120 µL of TfR1 P2 viruses. Growth media were analyzed 72 h post-infection. For time point expression analysis, 1 mL culture samples were taken from a 30 mL culture of infected cells, at different time points post-infection, as indicated.

Human cells: For antibody expression and secretion, two plasmids (for light and heavy chains) were co-transfected together simultaneously into cells. Antibodies were expressed in either adherent or suspension-grown HEK293 cells. Adherent cells were grown in Dulbecco’s modified Eagle’s medium (DMEM) supplemented with 10% fetal calf serum, penicillin-streptomycin, sodium pyruvate, nonessential amino acids (Biological Industries), and MycoZap (Lonza) in a humidified incubator at 37 °C, in a 5% CO_2_ controlled environment. Suspension-grown HEK293F cells were grown in FreeStyle medium (Gibco), in a shaking incubator, at 37 °C, in a controlled environment of 8% CO_2_, at 115 rpm. Transfections to adherent cells were performed in 80% confluent 10-cm dishes, using jetPEI (PolyPlus), with 5 µg of the light chain plasmid, and 5 µg of the heavy chain plasmid. Post-transfection, cells were transferred to a growth medium containing 2% serum. Growth media were collected 48–72 h post-transfection. Transfections to suspension-grown cells were performed at a cell density of 10^6^ cells/mL, using linear 40 kDa polyethylenimine (PEI) (Polysciences) at 3 mg of PEI per 1 mg of plasmid (0.5 mg of the light chain plasmid and 0.5 mg of the heavy chain plasmid) per 1 L of culture. Growth media were collected after 5–7 days.

### Sample preparation

In general, crude media were cleared from cells and insoluble debris by centrifugation, and then buffer exchanged into 1 M ammonium acetate. We found experimentally that performing 1–2 cycles of buffer exchange into high salt ammonium acetate efficiently replaces the bulk non-volatile compounds in the sample, and result in high quality spectra. In some instances, as detailed below, we performed an additional buffer exchange cycle, into a low concentration of ammonium acetate (150–200 mM).

*CBM3a and serum albumin: P. pastoris* cells were cleared from the medium by centrifugation at 14,000×*g* for 2 min. The growth medium was then supplemented with protease inhibitors (1 mM PMSF, 1 mM benzamidine, 1.4 μg/mL pepstatin A), aliquoted, snap- frozen in liquid nitrogen, and stored at −80 °C. On the day of the measurements, samples were thawed, and buffer exchanged 2–3 times into 1 M ammonium acetate, pH 7, using Micro Bio-Spin™ 6 Columns (Bio-Rad) and directly sprayed into the mass spectrometer.

For cofactor binding analysis, 30–50 μl buffer exchanged growth medium was incubated for 20 min at room temperature with 50 mM EGTA, and then buffer exchanged again into 0.2 M ammonium acetate, pH 7. Ca^2+^ treatments were carried out by adding 0.5 mM of calcium acetate to the growth medium.

TfR1: Insect cells were cleared from the medium by centrifugation at 1000×*g* for 5 min. The growth medium was then supplemented with protease inhibitors, as described, and cleared of insoluble materials by centrifugation at 10,000×*g* for 10 min. The supernatant was aliquoted, snap frozen in liquid nitrogen, and stored at −80 °C. On the day of the measurements, samples were thawed, and buffer exchanged 2–3 times into 1 M ammonium acetate, pH 7, and directly sprayed into the mass spectrometer. For deglycosylation experiments, 50–100 μL of buffer exchanged growth medium was incubated for 10 min at 50 °C in the presence of 1 µL of Rapid PNGaseF (New England Biolabs) and Rapid PNGase F buffer, and then buffer exchanged again into 1 M ammonium acetate. For glycan analyses, 50–100 μL of buffer exchanged growth medium was incubated for 5 h at 37 °C in the presence of 0.2 µL of PNGaseF (New England Biolabs), then directly sprayed into the instrument.

Antibodies: Growth media were cleared from cells by centrifugation at 600×*g* for 5 min, supplemented with 0.02% (w/v) sodium azide and 1 mM PMSF, and further clarified by centrifugation at 10,000×*g* for 15 min. Clarified media were aliquoted, snap frozen in liquid nitrogen and stored at −80 °C. On the day of the measurements, samples were thawed and buffer exchanged 2 times into 1 M ammonium acetate, pH 7. In order to reduce cysteine bonds, we tested different concentrations of the reducing agents DTT and TCEP, added directly to the growth medium, at different incubation times. We found that incubation of the medium of the G6^des13^ antibody in the presence of 20 mM TCEP for 4 h at 37 °C, followed by two consecutive buffer exchanges into 1 M and 150 mM ammonium acetate, respectively, gave the best results. For deglycosylation experiments, 50–100 µL of buffer exchanged growth medium was incubated for 10 min at 50 °C in the presence of 1 µL of Rapid PNGaseF (New England Biolabs) and Rapid PNGase F buffer, and then buffer exchanged again into 1 M and 150 mM ammonium acetate. For glycan analyses, 50–100 µL of buffer exchanged growth medium was incubated for 9 h at 37 °C in the presence of 0.2 µL of PNGaseF (New England Biolabs), then directly sprayed into the mass spectrometer.

To visualize proteins on gels, aliquots from different samples were mixed with 5× protein sample buffer, boiled for 5 min, and separated by SDS-PAGE. Gels were then stained with InstantBlue (Expedeon), or transferred to polyvinylidene difluoride membranes, and probed with a His Probe HRP-Conjugate (Thermo, 15165) at a dilution of 1:500, according to the manufacturer’s instructions, or an anti-GFP antibody (Abcam, ab290) at a dilution of 1:2500.

### Native mass spectrometry

Nano-electrospray ionization MS experiments were performed on two instrument platforms, a modified Q-Exactive Plus Orbitrap EMR^14^ (Thermo Fisher Scientific, Bremen, Germany) and a Synapt G2 instrument modified for high mass analysis (Waters, Hertfordshire, U.K.). All measurements were performed at least three times. Spectra recorded by the Orbitrap platform were converted to Masslynx-compatible files using the Waters DataBridge software. All spectra were analyzed using the MassLynx V4.2 SCN982 software (Waters, Hertfordshire, U.K.). Reported masses (shown in Daltons) and mass errors were calculated using MassLynx. Mobilograms were generated by the Driftscope^TM^ HDMS^TM^ V2.8 software (Waters, Hertfordshire, U.K.). Top-down proteomic analysis was performed using the ProSightPC v.3.0 software.

Typically, an aliquot of 2 μL protein solution was loaded into a gold-coated nano-electrospray ionization capillary prepared in-house, as previously described^39^, and sprayed into the instruments. The following experimental parameters were used on the Orbitrap platform: All spectra are shown without smoothing, and the instrument was calibrated externally, using cesium iodide. Conditions within the mass spectrometer were adjusted to preserve noncovalent interactions, and adjusted according to the type of protein measured. The source was operated in positive mode, capillary voltage was set to 1.7 kV, capillary temperature was 180 °C, and argon was used as the collision gas in the HCD cell. MS spectra were recorded at a resolution of 10,000–70,000, according to the complexity of the sample. Raw spectra were deconvoluted using Deconvolution 4 protein software (Thermo).

For CBM3a and albumin measurements, HCD voltage was set to 25 V and 85 V, respectively, at a trapping gas pressure setting of 1, which corresponds to an HV pressure of 9.2 × 10^–6^ mbar, and a UHV pressure of 6.3 × 10^–11^ mbar. Bent flatapole DC bias and axial gradient were set to 1.8 V and 10 V, respectively.

For TfR1 measurements, HCD voltage was set to 25 V, at a trapping gas pressure setting of 1.5, which corresponds to HV pressure of 9.8 × 10^–6^ mbar, and UHV pressure of 6.9 × 10^–11^ mbar. Bent flatapole DC bias and axial gradient were set to 1.8 V and 15 V, respectively.

For antibody measurements, HCD voltage was set to 50–100 V, at a trapping gas pressure setting of 3.9, which corresponds to HV pressure of 1.04 × 10^–4^ mbar, and UHV pressure of 2.35 × 10^–10^ mbar. Bent flatapole DC bias and axial gradient were set to 2 V and 25 V, respectively.

For the measurement of intact glycans, no HCD voltage was applied. Trapping gas was set to a pressure of 1, which corresponds to HV pressure of 9.2 × 10^–6^ mbar, and UHV pressure of 6.7 × 10^–11^ mbar. Bent flatapole DC bias and axial gradient were set to 1.8 V and 6 V, respectively.

The following experimental parameters were used on the Synapt G2 platform for IM-MS measurements: All spectra are shown with minimal smoothing, and the instrument was calibrated externally, using cesium iodide.

For CBM3a measurements, instrument parameters were set as follows: Capillary voltage of 1.5 kV, sampling cone 20 V, extraction cone 2 V. No trap and transfer collision energies were applied. Trap DC bias 45 V, and helium cell gas flow 120 mL/min. Nitrogen was used as the IMS gas, at a flow of 60 mL/min. IM wave velocity was set to 300 m/s, and wave height was set to 18, 19, or 20 V. Collision cross-sections were calculated for the +7 and +8 charge states of CBM3a.

For antibody measurements, instrument parameters were set as follows: Capillary voltage of 1.5 kV, sampling cone 10 V, extraction cone 2 V. Trap collision energy 20 V. No transfer collision energy was applied. Trap DC bias 45 V. Helium cell gas flow 120 mL/min. Nitrogen was used as the IMS gas, at a flow of 60 mL/min. IM wave velocity was set to 300 m/s, and wave height was set to 18, 19, or 20 V. Collision cross-sections were calculated for the +21, +22, and +23 charge states of the antibodies.

Proteins used for calibrations were: Ubiquitin (8.6 kDa); cytochrome-C (12.2 kDa); BSA (67.2 kDa); concanavalin A (103.1 kDa); alcohol dehydrogenase (149.3 kDa); and pyruvate kinase (227.0 kDa), all purchased from Sigma. Calibrants were dissolved in 200 mM ammonium acetate solutions to retain a native-like conformation. All measurements were conducted on the same day; the only parameters modified for the test and calibrating proteins were capillary voltages. CCS values were calculated using Driftscope CCS calculation software (V2.8, Waters Corp., Manchester, U.K.) according to ref. ^40^. The theoretical CCS value for CBM3a (PDB Entry 4JO5) was calculated using the Driftscope Projection Approximation algorithm (Waters).

For quantification and dilution experiments, we collected growth media from non expressing cells from the three different expression systems, yeast, insect and human cells, cultured for 24–48 h, and buffer exchanged twice into 1 M ammonium acetate. Protein concentration was determined using the Bio-Rad Protein Assay Dye Reagent (500–0006). For quantification experiments, 4 µL of the buffer exchanged growth media were mixed with 1 µL of BSA (Sigma A-4378) at different concentrations, as indicated. Spectra were recorded on an Orbitrap instrument, as described for serum albumin. For each mass spectrum, peak intensities of the three major charge states of BSA (+14, +15, and +16) were summed and divided by the intensity of selected background peaks, which served as references. The following reference proteins were selected: 2088, 2560, and the sum of *m*/*z* 4376 and 4668 *m*/*z* (corresponding to a protein with a mass of 70,000 Da) in the yeast, insect and human growth media, respectively. The relative values obtained were plotted against known BSA concentrations. For dilution experiments, yeast, insect and human cell cultures containing 20 µM BSA were serially diluted with 150 mM ammonium acetate and spectra were recorded on the Orbitrap instrument, as described above. Each experiment was performed at least three times.

## Electronic supplementary material

Description of Additional Supplementary Files

Supplementary Data 2

Supplementary Data 1

Supplementary Information

## Data Availability

The datasets generated during the current study are available from the corresponding author on reasonable request.
