# Supplementary material for: Rapid characterization of secreted recombinant proteins by native mass spectrometry
Source: Commun Biol. 2018 Dec 3;1:213. doi: 10.1038/s42003-018-0231-3 (PMC6277423; doi:10.1038/s42003-018-0231-3)
Supplement: Supplementary file 1 — Description of Additional Supplementary Files [file 42003_2018_231_MOESM1_ESM.docx]

**Description of Additional Supplementary Files**

**File Name**: Supplementary Data 1

**Description**: -Original data used to generate graphs in Figure 3.

**File Name**: Supplementary Data 2

**Description**: Original mean source data used to generate graphs in Figure 4.
