# Supplementary material for: Rapid characterization of secreted recombinant proteins by native mass spectrometry
Source: Commun Biol. 2018 Dec 3;1:213. doi: 10.1038/s42003-018-0231-3 (PMC6277423; doi:10.1038/s42003-018-0231-3)
Supplement: Supplementary file 4 — Supplementary Information [file 42003_2018_231_MOESM4_ESM.pdf]

Supplementary Figures

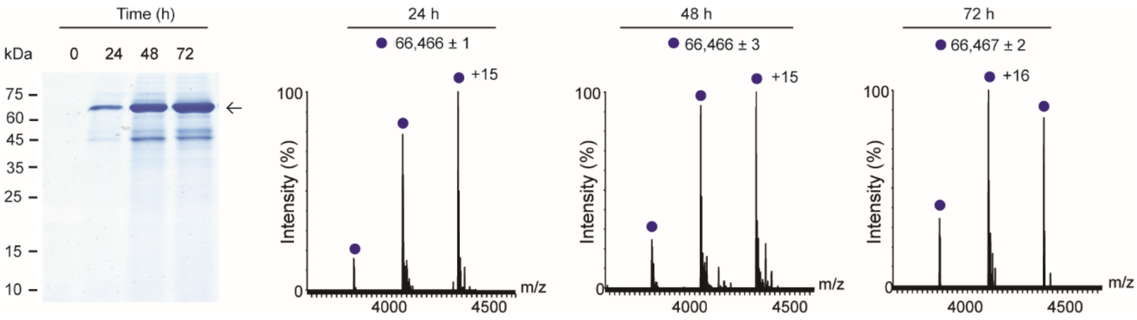

Supplementary Figure 1. Serum albumin can already be detected in the growth medium, 24h post-induction. SDS-PAGE and the corresponding mass spectra show the expression rates of albumin over a 72-hour period following induction. The band corresponding to albumin is denoted with an arrow (the uncropped gel image can be seen in supplementary figure 11D). Peaks corresponding to albumin can be detected 24 hours post-methanol induction. The narrow charge state distribution indicates that the protein is expressed in a soluble and folded form.

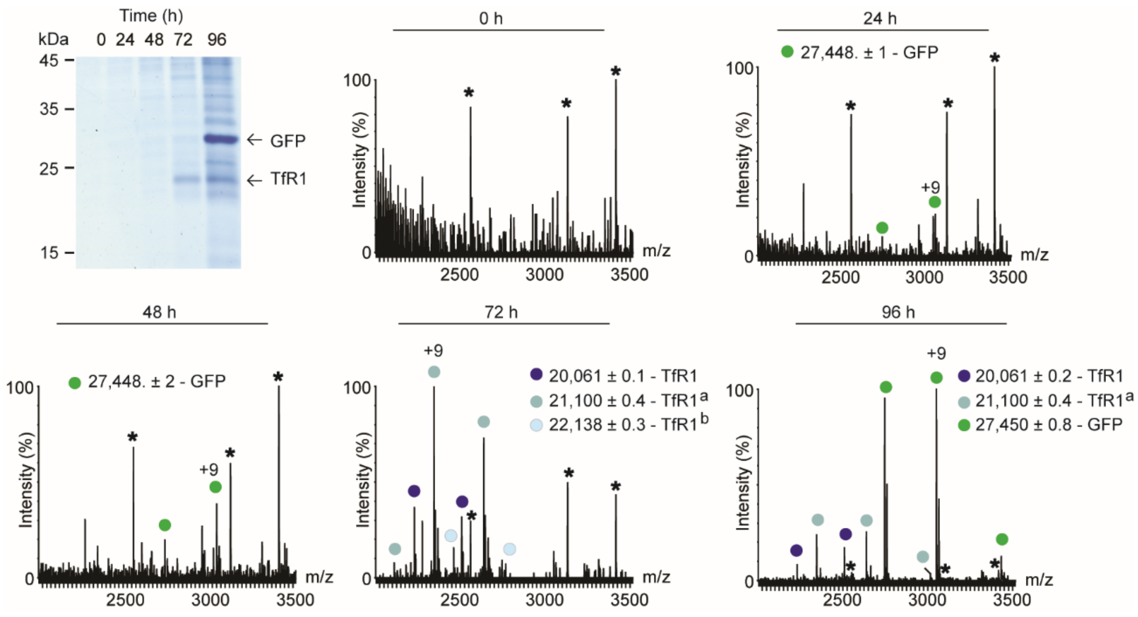

Supplementary Figure 2. Time course analysis of TfR1 expression by direct MS analysis of insect cell growth medium.

SDS-PAGE and direct-MS analysis of secreted TfR1 over a time span of 96 hours post infection. Bands corresponding to TfR1 and GFP are denoted with arrows (the uncropped gel image can be seen in supplementary figure 11E). GFP (green circles) can be detected as early as 24 hours after expression is initiated. The different proteoforms of the protein, intact TfR1, as well as TfR1 modified with GlcNac$_2$Man$_3$Fuc$_1$ (TfR1[a]) and with two GlcNac$_2$Man$_3$Fuc$_1$ saccharides (TfR1[b]), start to appear after 72 hours. At 96 hours, the GFP charge series becomes the dominant series in the spectrum, probably due to increased cell death, which results in spillage of the cellular contents (including the internally expressed GFP) into the growth medium. Background protein signals from the growth medium are denoted with asterisks. The reduced contribution of the background peaks correlates with TfR1 accumulation.

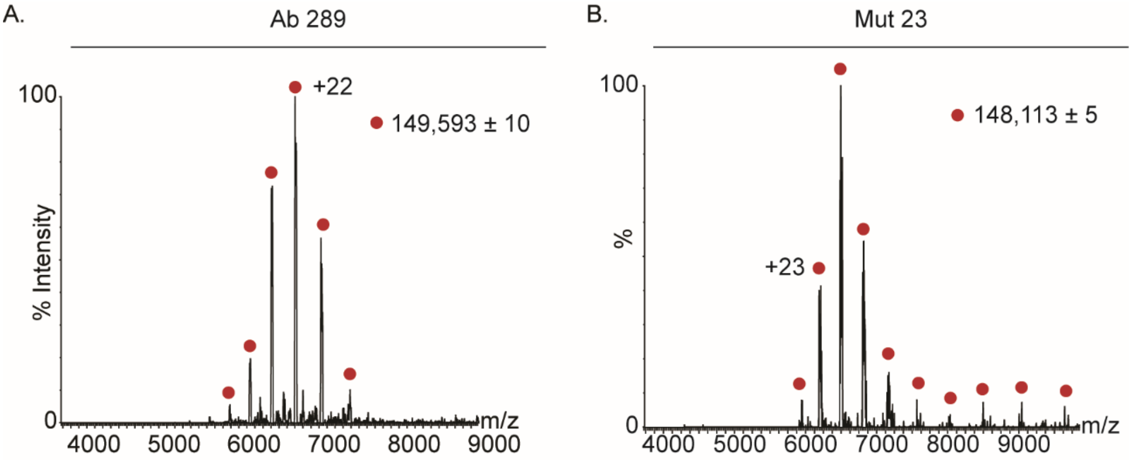

Supplementary Figure 3. Rapid characterization of recombinant antibodies, directly from the growth medium of secreting human cells.

Direct-MS spectrum recorded for the **(A)** 289 and **(B)** Mut23 antibodies, produced in adherent and suspension-grown HEK293 cells, respectively.

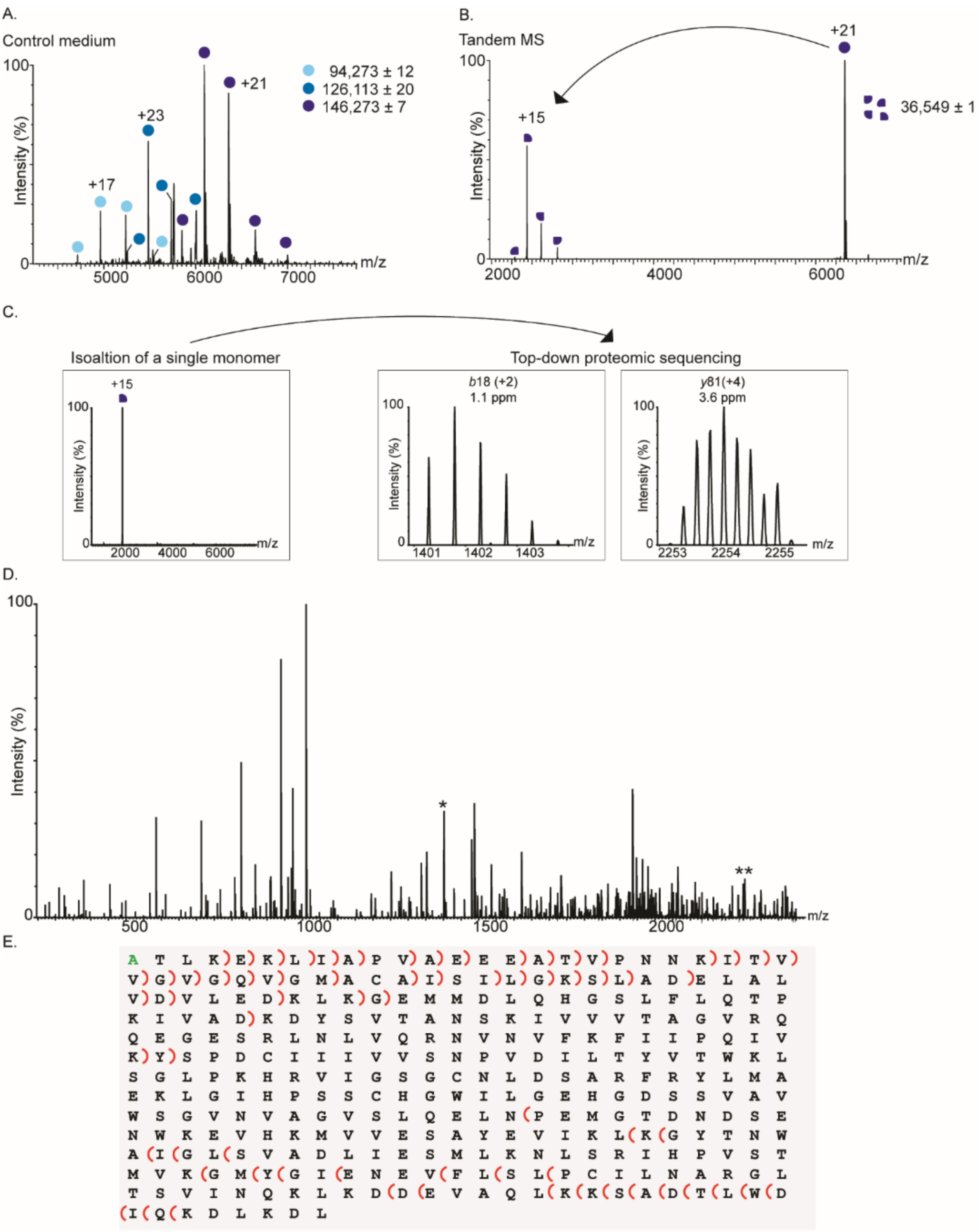

Supplementary Figure 4. Top-down proteomic analysis indicates that the major background protein found in the medium of HEK293F cells is lactate dehydrogenase. (A) Native MS analysis of the growth medium from non-transfected cells reveals the presence of different proteins. **(B)** Tandem MS analysis of the +21 charge state, corresponding to the 146 kDa protein indicated that it is

a tetramer, composed of monomers bearing a mass of 36.5 kDa. **(C)** In order to define the identify of this protein, pesudo-MS^3 analysis was performed. The +16 charge state of the 36.5 kDa monomer was isolated, and fragmented within the HCD cell. Two representative fragments, are shown. **(D)** Spectrum showing all the generated fragments. One and two asterisks label the *b18* and *y81* ions that are shown in **(C)**, respectively. (E) In total, 68 fragments were matched to the protein sequence of lactate dehydrogenase B chain, resulting in a 20% coverage of the protein. The identified protein is missing the N-terminal methionine and consists of an N-terminal acetylation.

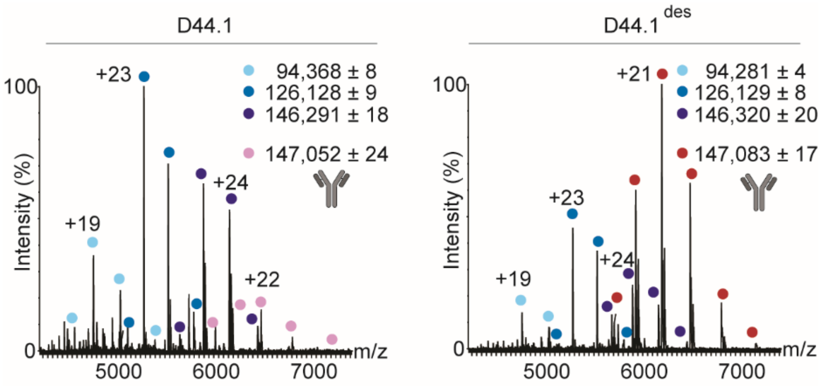

Supplementary Figure 5. The designed D44.1^{des} antibody accumulates to higher levels in the growth medium of suspension-grown HEK293 cells, compared to D44.1, its wild-type counterpart.

Expression levels of the secreted D44.1 and its designed variant D44.1^{des} were examined directly from the growth medium. Comparison of the generated spectra indicate that D44.1 accumulates to a lesser extent than D44.1^{des}. Background media proteins are labeled with different shades of blue circles. The antibody cartoon labels the measured mass of the antibody.

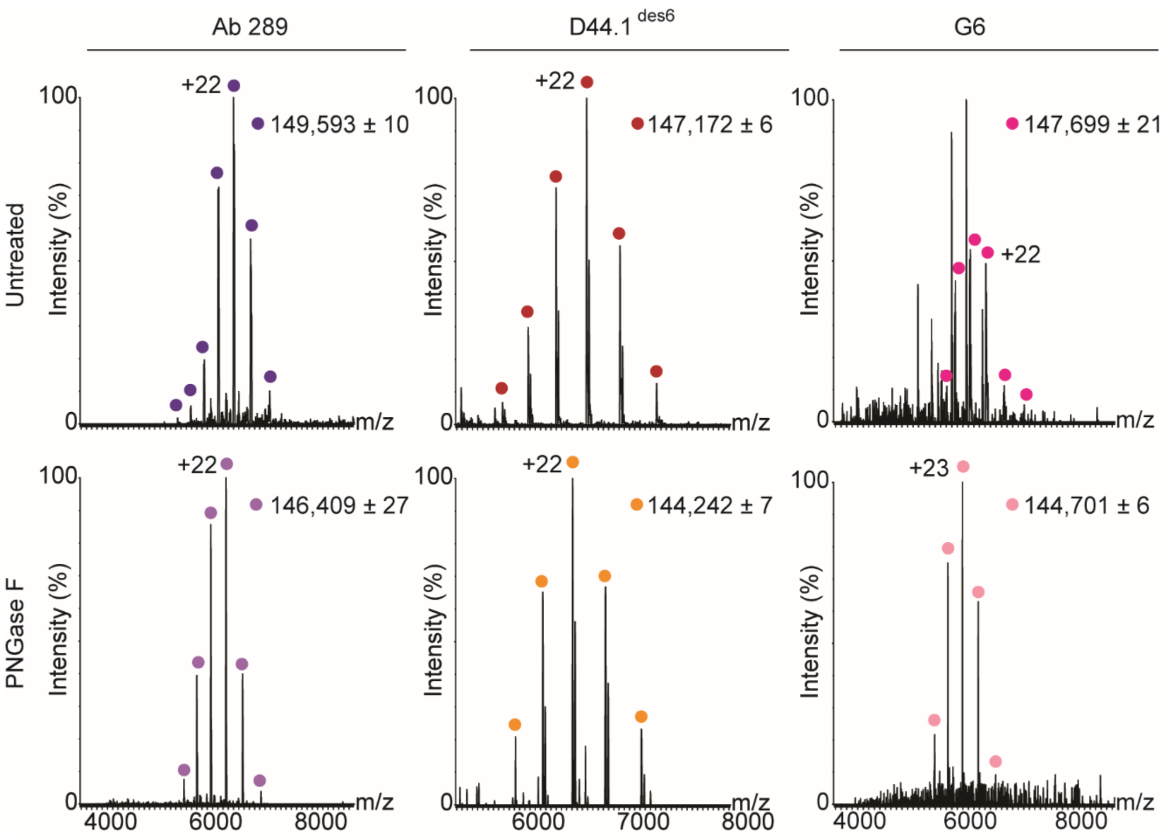

Supplementary Figure 6. Deglycosylation of antibodies secreted from adherent and suspension-grown HEK293.

Treatment of the growth medium of different antibodies with PNGase F resulted in removal of their associated glycans, with a corresponding reduction in mass of approximately 3 kDa. Minor subpopulations of partially or fully glycosylated antibodies were also detected in the treated samples.

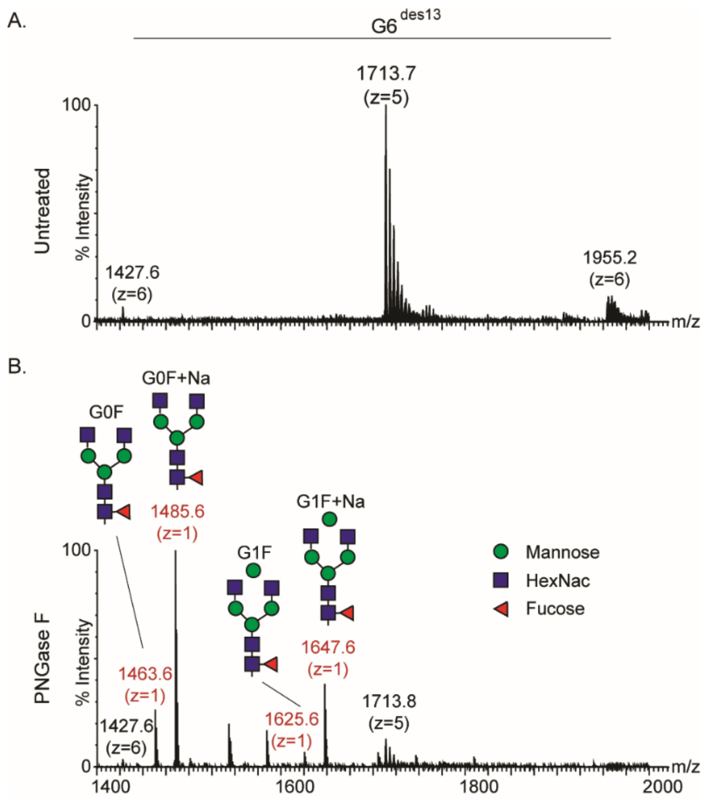

Supplementary Figure 7. Analysis of released glycans following PNGase F treatment.

Direct-MS spectra were acquired prior to **(A)** and following treatment with PNGase F **(B)**. Data acquired indicate the release of two glycans, corresponding in mass to G0F and G1F. Both sodiated and non-sodiated forms of the glycans were detected. The m/z values of the single-charged glycans are labeled in red, and those of multiple-charged contaminant peptides are labeled in black.

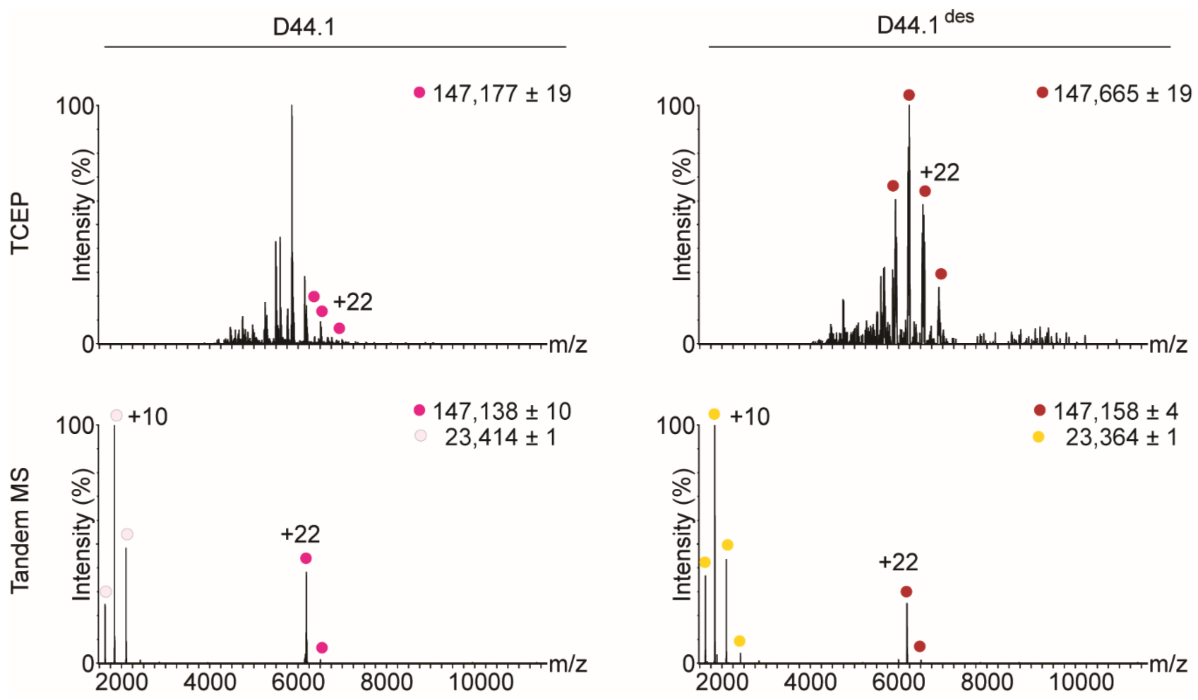

Supplementary Figure 8. Tandem MS of the reduced D44.1 and D44.1^{des} antibodies secreted from suspension-grown HEK293 cells.

Antibodies were reduced by addition of TCEP to the growth medium, resulting in the dissociation of the light chains, as demonstrated by tandem MS analyses. The measured masses of the dissociated light chains are smaller than their theoretical masses by 4 Da, indicating that TCEP did not reduce the two intermolecular sulfur bridges within the light chains. In addition, the close agreement between the measured and calculated mass of the light chains implies that the light chains are not glycosylated; rather, the heavy chains are modified with glycans.

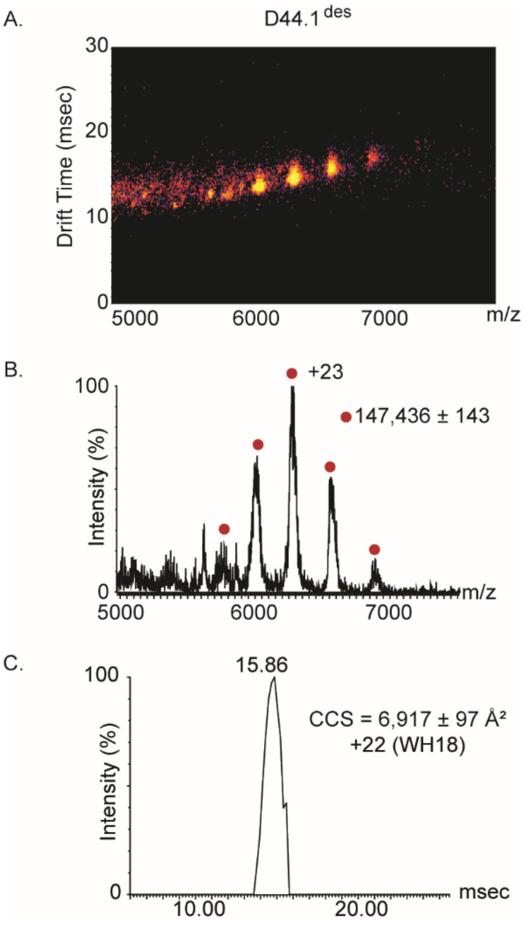

Supplementary Figure 9. Collision cross-section measurement of the D44.1^{des} antibody.

(A) A three-dimensional IM-MS spectrum was measured for the D44.1^{des} antibody. **(B)** A representative two-dimensional plot of m/z versus intensity of D44.1^{des}. **(C)** Representative ion mobility arrival time distribution for the +22 charge state of the antibody, measured at a wave height (WH) of 18 V, is displayed alongside the calculated CCS value.

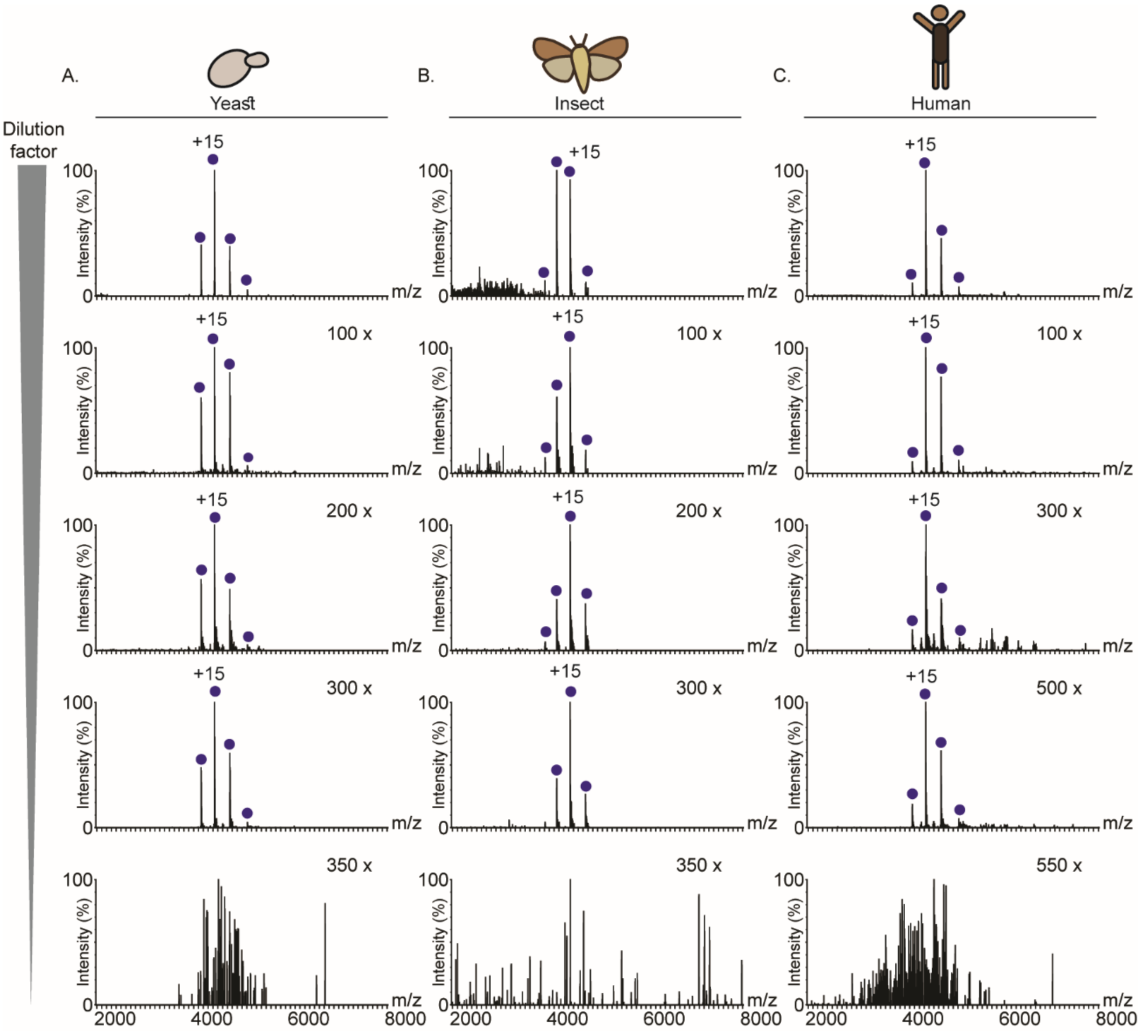

Supplementary Figure 10. Dilution of crude growth media samples improves signal-to-noise ratio.

In order to explore the effect of sample dilution, we serially diluted yeast **(A)**, insect **(B)** and human cells **(C)** growth media containing 20 µM of externally-added BSA (dark blue circles). Samples were diluted until the detection limit was reached. In addition to the improved signal-to noise ratio that the diluted spectra benefited from, we noticed that quantities below 100 nM of BSA can be detected in the three growth media.

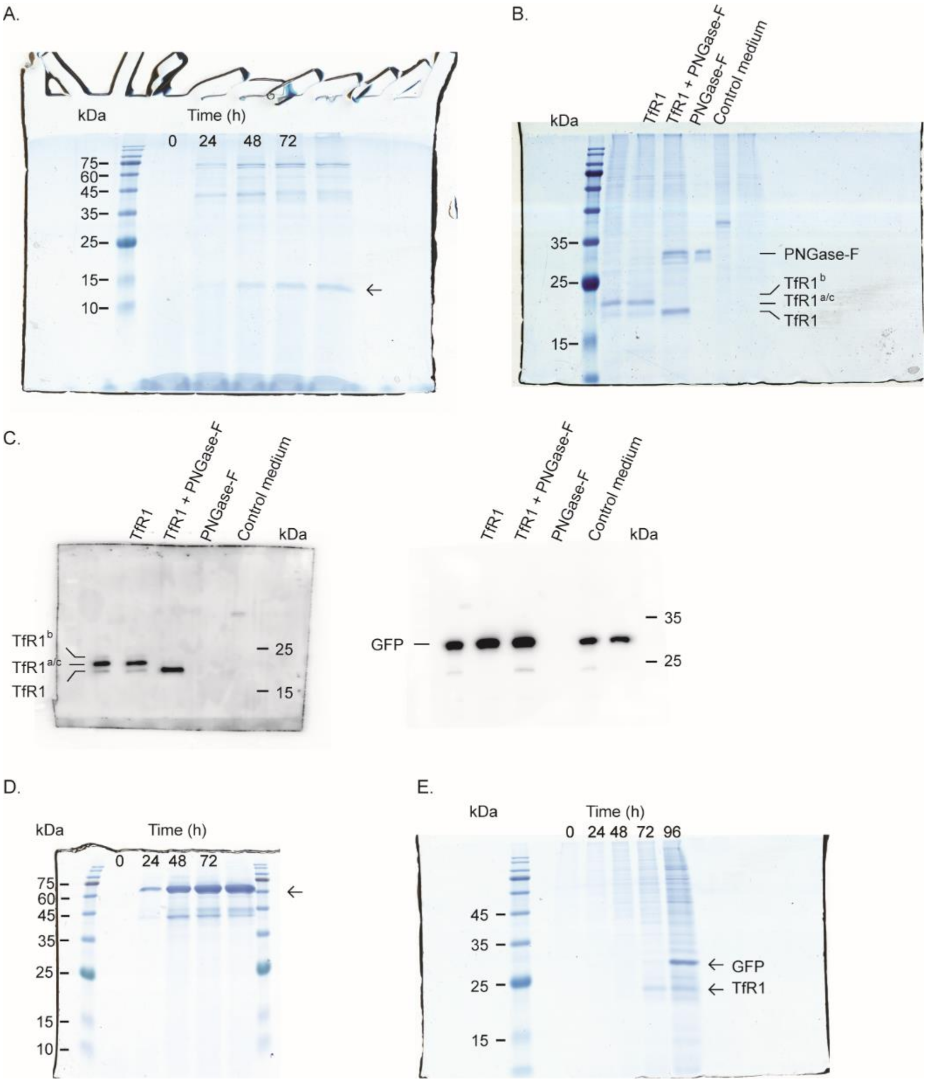

Supplementary Figure 11. Uncropped gel and blot images.

Full, uncropped images of the gels and blots from Figure 1A **(A)**, Figure 2C **(B)**, Figure 2D **(C)**, supplementary Figure 1 **(D)** and supplementary Figure 2 (E), are shown.

Glycans and glycan fragments of TfR1

	Glycan	Theoretical mass	Actual mass	Δ (Da)	Mass error (ppm)
MS	$[GlcNac_2Man_3Fuc_1]$+reducing end+H	1057.3935	1057.3933	0.0002	-0.17
	$[GlcNac_2Man_3]$+reducing end+H	911.3356	911.3387	-0.0031	3.42
MS/MS @ 1057 m/z	$[GlcNac_2Man_3Fuc_1]$+reducing end+H	1057.3935	1057.3940	-0.0005	0.49
	$[GlcNac_2Man_3Fuc_1]$+H	1039.3829	1039.3792	0.0037	-3.58
	$[GlcNac_2Man_3]$ + reducing end+H	911.3356	911.3324	0.0032	-3.49
	$[GlcNac_2Man_3]$+H	893.3250	893.3231	0.0019	-2.15
	$[GlcNac_2Man_3Fuc_1]$+H	836.3035	836.3020	0.0015	-1.82
	$[GlcNac_2Man_2]$+reducing end+H	749.2828	749.2811	0.0017	-2.24
	$[GlcNac_1Man_3]$+reducing end+H	708.2562	708.2548	0.0014	-1.95
	$[GlcNac_2Man_1]$+reducing end+H	587.2300	587.2271	0.0029	-4.90
	$[GlcNac_1Man_2]$+H	528.1928	528.1931	-0.0003	0.52
	$[GlcNac_2]$+reducing end+H	425.1772	425.1767	0.0005	-1.13
	$[Man_2]$+reducing end+H	325.1134	325.1129	0.0005	-1.40
	$[GlcNac_1]$+H	204.0872	204.0866	0.0006	-3.06
MS/MS @ 911 m/z	$[GlcNac_2Man_3]$+reducing end+H	911.3356	911.3337	0.0019	-2.06
	$[GlcNac_2Man_3]$+H	893.3250	893.3243	0.0007	-0.81
	$[GlcNac_2Man_2]$+reducing end+H	749.2828	749.2819	0.0009	-1.17
	$[GlcNac_1Man_3]$+reducing end+H	708.2562	708.2544	0.0018	-2.51
	$[GlcNac_2Man_1]$+reducing end+H	587.2300	587.2308	-0.0008	1.40
	$[GlcNac_1Man_2]$+H	528.1928	528.1931	-0.0003	0.52
	$[GlcNac_2]$+reducing end+H	425.1772	425.1767	0.0005	-1.13
	$[GlcNac_1Man_1]$+H	366.1400	366.1397	0.0003	-0.89
	$[GlcNac_1]$+H	204.0872	204.0871	0.0001	-0.61

Supplementary Table 1. List of assigned glycans and glycan fragments identified for TfR1, following tandem MS (MS/MS). The 911 and 1057 m/z ions were selected and fragmented within the HCD cell. The table includes the theoretical masses, measured masses, mass differences (Δ) and mass measurement errors (ppm).
